# Dimensions of Subjective Well-Being

**DOI:** 10.1007/s11205-014-0753-0

**Published:** 2014-09-13

**Authors:** Arie Kapteyn, Jinkook Lee, Caroline Tassot, Hana Vonkova, Gema Zamarro

**Affiliations:** 1 Center for Economic and Social Research (CESR), University of Southern California (USC), Los Angeles, CA USA; 2 Davis School of Gerontology and CESR, USC, and RAND Corporation, Los Angeles, CA USA; 3 Faculty of Education and First Faculty of Medicine, Charles University in Prague, Prague, Czech Republic

**Keywords:** Subjective well-being, Response scales, Life satisfaction

## Abstract

We use two waves of a population based survey (the RAND American Life Panel) to investigate the relations between various evaluative and experienced well-being measures based on the English Longitudinal Study of Aging, the Gallup Wellbeing Index, and a 12-item hedonic well-being module of the Health and Retirement Study. In a randomized set-up we administered several versions of the survey with different response scales. Using factor analysis, we find that all evaluative measures load on the same factor, but the positive and negative experienced affect measures load on different factors. We find evidence of an effect of response scales on both the estimated number of underlying factors and their relations with demographics. We conclude that finer response scales allowing more nuanced answers offer more reliability. The relation of evaluative and experienced measures with demographics are very different; perhaps the most striking aspect is the lack of a consistent relation of experienced well-being measures with income, while evaluative well-being is strongly positively related with income.

## Introduction

Recent years have shown a proliferation of studies using various measures of happiness and life satisfaction, making it perhaps one of the most stimulating new developments in the social sciences (Frey and Stutzer 2005; Kahneman et al. 2004a). Recent government initiatives in countries such as France, through the Commission on the Measurement of Economic Performance and Social Progress (Stiglitz et al. 2009), the United Kingdom, through the Office of National Statistics (Dolan et al. 2011), or the United States, with Federal Reserve Chairman Ben Bernanke declaring his interest in finding better measurements of Americans’ well-being (Rugaber 2012), have further spurred a debate in the scientific community.

The majority of findings on subjective well-being are based on evidence from global life satisfaction measures used in large scale surveys. Throughout the literature, these findings have raised methodological concerns, as minor events and moods may influence responses to those questions, though there is a lack of consensus regarding the extent of such context effects (Schwarz and Strack 1991, 1999; Schimmack and Oishi 2005; Eid and Diener 2004). Global life satisfaction scales have produced widely conflicting findings. A prominent example is the so-called Easterlin paradox, where some authors found that happiness levels across countries show no relationship with the level of economic development of a country (Easterlin 1974, 1995), while others found a monotonic relationship between economic development and subjective well-being (Deaton 2008; Kahneman and Deaton 2010; Stevenson and Wolfers 2008).

Apart from global life satisfaction, other alternative subjective well-being measures have also been proposed in the literature. Although their classification has been somewhat controversial (Kahneman and Riis 2005), most of the psychology literature thus far has conceptualized subjective well-being either as the evaluation of life satisfaction/dissatisfaction (evaluative well-being measures) or as the combination of experienced affect—range of emotions from joy to misery—(experienced well-being measures). These two types of well-being measures are the focus of this paper. We also added, however, a third type of measure, a ‘eudemonic’ category to our study to fit the United Kingdom’s Office for National Statistics classification (Dolan et al. 2011) as will be explained below.

Broadly, the evaluative component of subjective well-being includes the elicitation of a respondent’s global subjective evaluation of his or her life, where the evaluation can also be limited to specific domains of life, such as satisfaction with work, family life, or health (Dolan et al. 2011). Typically, these questions are formulated as single item self-reports, formulated for example as “All things considered, how satisfied are you with your life as a whole these days?” or “Taken all together, would you say that you are very happy, pretty happy, or not too happy?” (Krueger and Schkade 2008). More recent surveys however have included multiple questions eliciting evaluative well-being. Perhaps most widely used among the latter is the Satisfaction with Life Scale (SWLS), which measures life satisfaction by asking respondents to report their level of agreement with five statements on a seven-point response scale from strongly disagree to strongly agree (Diener 2000; Diener et al. 1985). Though the response time to single global life satisfaction questions is lower than for multi-item measures, as one would expect, the latter appears to be more reliable. Typically, it is assumed that life satisfaction should not show large variation within short periods of time. When evaluating the reliability of evaluative measurements over time, the SWLS displays an estimated reliability—that is, the correlation across waves—of about 0.8 (Eid and Diener 2004; Krueger and Schkade 2008), compared with single item global life satisfaction measures that have an estimated reliability of about 0.60 (Andrews and Whithey 1976; Krueger and Schkade 2008). Evaluative questions are the most frequently used survey items within the field of subjective well-being (Kahneman and Krueger 2006). For instance, most of the large longitudinal ageing surveys have included this type of life satisfaction measures in their questionnaires. The Health and Retirement Study (HRS) and the English Longitudinal Study of Aging (ELSA) include Diener’s five-item SWLS (Diener et al. 1985). The HRS and the Survey of Health, Aging and Retirement in Europe (SHARE) include a single item overall life satisfaction question in their core interviews. Other measures of evaluative well-being often used in studies include Campbell’s domain-specific life satisfaction (Campbell et al. 1976) used in the Gallup Wellbeing Index: Standard of Living and Personal Life, and the Cantril Self-Anchoring Striving Scale (Cantril 1965), often referred as Cantril ladder, used by the Gallup poll and the OECD.

While evaluative life satisfaction questions have been widely used, their meaning and research application remain a matter of debate. Life satisfaction is a global retrospective judgment, cognitively demanding, and likely constructed only when asked. Respondents may thus base their answer on heuristics, their current mood and memory (Kahneman and Krueger 2006; Schwarz and Strack 1999). The difficulty of investigating such effects is made obvious by Lucas and Lawless (2013), who note that while weather has often been found to affect the mood and life satisfaction of respondents, this may have been the result of different climates, or time of the year, as they find no effect of weather itself in a large scale study. In contrast to evaluative subjective well-being measures that require an evaluative judgment from respondents, experienced well-being measures focus on how respondents are feeling (positive and negative affect) at a specific point in time. These experienced measures correspond to a rather Benthamite view of well-being, in that the latter depends entirely on individuals’ feelings, though the list of feelings used in surveys is usually not limited to pleasure and pain (Dolan et al. 2011). Experienced well-being is thus based on real-time affect measurements (Kahneman et al. 2006).

Ecological Momentary Assessment (EMA) aims at a “repeated collection of real-time data on subjects’ behavior and experience in their natural environments” (Shiffman et al. 2008, p. 3). The term EMA was coined by Stone and Shiffman (1994). Such data can be collected by a variety of methods, including time based designs whereby for instance subjects are prompted at random intervals to record their activities or mood, or event based designs whereby subjects are asked to provide information after specific events. Although EMA can be applied in pretty much any domain of human activity that one wants to measure in real time and in individuals’ natural environment, our interest here is primarily in the measurement of affect. Frequent measurements permit the detection of variation in affect over time and during particular activities, and thus yield high reliability and validity of measures (Csikszentmihalyi and Hunter 2003). EMA may be costly however, and may place a high burden on respondents (Kahneman and Riis 2005).

The Day Reconstruction Method (DRM) has been developed to offer some of the advantages of EMA while being more practical, by combining a time-use survey with questions about the previous day (Kahneman et al. 2004b). DRM surveys can include details such as the type of activity, location, presence of other individuals and experienced affect for all activities listed by a respondent in his diary, or only for a subset, e.g. three randomized times or activities throughout the day, as the Princeton Affect Time Use Survey (PATS) or the American Time Use Survey (ATUS) have implemented. While the DRM involves the retrospective report on an emotional state, this survey design targets accurate recall, by leading respondents to retrieve specific episodes and emotions from memory (Kahneman et al. 2004a). DRM is in some sense more complete than EMA, as it attempts full coverage of the day, whereas EMA samples several moments during the day. Studies have validated the results obtained through the DRM by comparing them with experience sampling methods (Kahneman and Krueger 2006). Other surveys, such as the Gallup World and Daily Polls aim at measuring experienced well-being simply by asking respondents about emotions experienced during the whole previous day; this then elicits emotions aggregated over many episodes during a day.^1^

Throughout the literature, the complementarity of evaluative and experienced measures of well-being is explained by the fact that both measures are likely correlated, though remaining empirically and conceptually different (Kahneman and Riis 2005). However, more research is needed to understand how the concepts experienced well-being measures are capturing differ from those captured by evaluative measures. Comparing these two types of measures is one of the objectives of this paper.

Finally, the last category of well-being measures we will consider in this paper refers to “eudemonic” survey items. Eudemonic measures refer to the existence of underlying psychological needs, encompassing various dimensions of wellness, such as autonomy, personal growth, or purpose in life, which contribute towards well-being independently of any positive affect they may convey (Dolan et al. 2011; Ryff and Keyes 1995). Ryff presents evidence of a certain degree of convergence between these “theory-guided” eudemonic well-being measures with the commonly used life satisfaction measures (Dolan et al. 2011; Ryff 1989). The question “Overall, to what extent do you feel that the things you do in your life are worthwhile?” is an example of eudemonic measure currently used by the Office of National Statistics in the UK (Dolan et al. 2011).

Overall, as pointed out by Krueger and Schkade (2008), relatively little attention has been paid to the reliability of experienced measures. While each existing measure of subjective well-being appears to show some evidence of validity^2^ and to capture distinct dimensions (National Research Council 2013), the differences between the measures of well-being have not been explored systematically. This paper aims at filling these gaps in the literature by studying the results of two waves of well-being data we collected in the RAND American Life Panel (ALP). In particular, we designed two experimental modules that were fielded in the ALP including some of the evaluative and eudemonic well-being measures described above, as well as a number of experienced measures. Our objective when choosing the measures for our questionnaires was to represent common well-being measures, often used in existing studies, and with different time requirements for the respondents, in order to be able to compare the concepts they are capturing. Another important comparison we study is the use of different response scales for the elicitation of well-being measures. Although the concepts asked in the different measures are in some cases the same, measures differ in the response scales used and so, we will study the correspondence across these different response scales. Results of this analysis will be useful to inform studies that aim at including these different measures.

The remainder of the paper is structured as follows. The next section describes the data we have collected and the experiment we have designed and implemented. Section 3 provides descriptive statistics as well as measures of reliability for various subjective well-being measures. In Sect. 4 we use factor analysis to explore the relation between those measures. Section 5 focuses on the effect of different response scales on the dimensionality of subjective well-being found when applying factor analysis. Section 6 compares how evaluative and experienced well-being measures differ in how they correlate with demographics. Section 7 concludes.

## Data and Experiment

### The RAND American Life Panel (ALP)

To conduct this research, we use data collected in the RAND ALP. At the time of the survey, the ALP consisted of approximately 5,500 respondents ages 18 and over who were interviewed periodically over the Internet. Respondents do not need Internet access to participate, although the majority of the panel members have their own Internet access. The remaining panel members (approximately 10 % of the sample) have been provided Internet access by RAND through the provision of a laptop or a Microsoft TV2 and/or an Internet subscription, eliminating the bias found in many Internet surveys that include only computer users (Chang and Krosnick 2009; Yeager et al. 2011). The TV2 is an Internet player that allows respondents to open email accounts and browse the Internet. Sampling weights are also provided by the ALP to adjust for sample selection. Upon joining the panel, respondents complete an initial survey collecting individual socio-demographic information, work history and household composition information. They are asked to update their background information every quarter. About once or twice a month, respondents receive an email with a request to fill out a questionnaire. Response rates average 70–80 %. Since January 2006, researchers have fielded over 300 surveys, and published papers using these data on a wide variety of topics, for instance subjective probabilities and expectations (Delavande and Rohwedder 2008; Manski and Molinari 2010), life satisfaction (Kapteyn et al. 2010), financial literacy (Bruine de Bruin et al. 2010; Fonseca et al. 2012; Lusardi and Mitchell 2007), and Presidential election polling (Gutsche et al. 2014).

Apart from its flexibility and cost effectiveness in collecting new data, an important advantage of the ALP is that it also allows for experimentation, e.g. by administering different surveys or different tasks to randomly selected subgroups. We make use of this feature in this paper by designing two experimental modules that were administered in the ALP. The first module was administered from the beginning of May 2012 until July 2012, while the second module started to be administered at the end of May 2012 and was in the field until early August 2012. 4,339 respondents answered our module for the first wave out of 5,495 eligible respondents, resulting in a response rate of 79 %. Respondents who completed the first wave were then invited to answer questions in the second wave. Out of 4,336 eligible respondents (three respondents of the first wave were not available for the second wave), 4,031 respondents answered the module for the second wave, resulting in a response rate of 93.3 %. The following sections describe the well-being measures collected in these modules as well as the experiment that we designed and implemented.

**Fig. 1:**
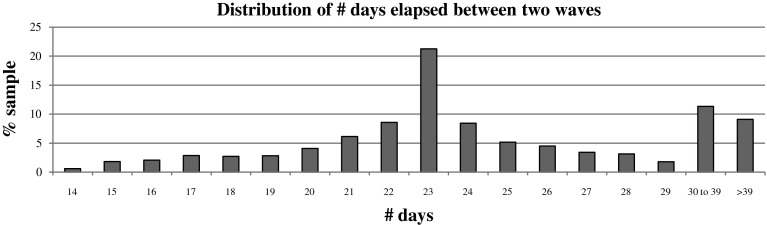
Time gap in responses between first and second wave

Finally, in administering the two modules in the ALP, we made sure that there would be at least 2 weeks between the waves. That is, a respondent would only be eligible for answering the questions in the second wave at least 2 weeks after this respondent had responded to the first wave. Respondents can answer question at a time that is convenient to them and as a result the time gap between the first and second waves may vary substantially. Figure 1 shows a distribution (in days) of the time gap between waves for the respondents in our sample. As per the protocol, the time gap is at least 14 days, with a very long tail, reflecting the fact that we kept the second wave in the field for a long time. The mean time gap between waves is 26.6 days with a standard deviation of 10.5 days. The peaks at 23 days and 30–39 days reflect email reminders to panel members, which had a noticeable effect on number of responses.

### Well-Being Measures in Our Questionnaires

In the two modules we fielded in the ALP, we administered four sets of evaluative well-being measures and three sets of experienced well-being measures.^3^ The evaluative well-being measures in our modules include the following: Diener’s five-item SWLS (Diener et al. 1985), in exactly the same form as it is included in the HRS and the ELSA; a single item overall life satisfaction question, identical to the one included in the SHARE; Campbell’s domain-specific life satisfaction (Campbell et al. 1976) used in the Gallup Wellbeing Index: Standard of Living and Personal Life, and the Cantril Self-Anchoring Striving Scale (Cantril 1965), often referred to as Cantril ladder, used by the Gallup poll and the OECD. In addition to these, we also included four questions from ELSA based on those collected by the UK Office of National Statistics (ONS) which comprise one evaluative life satisfaction question, one eudemonic question and two experienced well-being questions related to feelings of happiness and anxiety during the previous day. Although two of the ONS–ELSA questions are experienced well-being questions, in our experiment they are included in the evaluative measures group, as we seek to maintain a questionnaire structure as close to the original ONS questionnaire as possible. We will see however that in the analyses these questions behave differently than the evaluative measures, as one would expect.

Our ALP modules also included three sets of experienced well-being measures to be compared with the evaluative well-being measures described above, as well as among themselves. Our first set of experienced well-being measures comes from ELSA’s simplified version of the DRM collecting information about activities in the last day and how individuals felt when doing these activities. Our second group of experienced questions is based on the Gallup-Healthways well-being index. These questions collect information on a number of measures capturing positive and negative affect experienced yesterday. Finally, we also included questions from the so called HWB12, a newly developed experienced well-being measure by Smith and Stone (2011), which has been included in the 2012 wave of HRS. The HWB12 is a measure of 12 overall experiences of hedonic well-being referring to the previous day. The authors recommend asking wake and sleep times as a minimal check that participants focus attention on remembering the previous day and so, we also did. Finally, in order to facilitate the crosswalk across different experienced measures we added different sets of additional questions to each of the experienced measures included in our questionnaire as is explained in more detail in the following sections.

### Experiment

We fielded two waves of the ALP where we administered four evaluative well-being measures and three sets of experienced well-being measures. All evaluative well-being questions were asked in both waves.^4^ The experienced well-being measures show considerable overlap in the adjectives used in constructing the measures. To avoid contamination of responses within a wave, respondents answer only one set of experienced well-being measures in each wave, randomly assigned. Since there are only two waves, no one responds to all three experienced measures. We do make sure however that all possible combinations of experienced measures occur across the two waves. To be more precise, respondents are randomized into one of nine different groups for the experienced well-being measures: group 1–1 for example will see the Gallup questionnaire in both waves, while group 2–3 will see the ELSA questionnaire in the first wave, and the HWB-12 questionnaire in the second wave. This will apply for all combinations, i.e. 1–1, 2–2, 3–3, 1–2, 1–3, 2–1, 2–3, 3–1, 3–2.

As shown in the “Appendix”, for each set of experienced measures, respondents get a number of additional questions. The reason for this is as follows. The experienced measures differ in a number of ways. These include differences in the list of included items and differences in response scales. To be able to isolate the effects of differences in items and differences in response scales, we have added items to each of the experienced measures such that in each case a respondent answers exactly the same number of items. This allows us to look at both the effect of response scales (the different measures have different response scales, but the respondent answers the same number of items for every response scale) and the effect of the item choice (we can compare results with and without additional items; the additional items always come after the original set of items).

## Descriptive Statistics

Table 1 shows the response duration of different well-being measures we collected for the modules included in the ALP. Since respondents don’t have to take a survey in one sitting, total survey times sometimes may seem extremely long. To exclude such cases we omit observations for which total time exceeds 30 min (taking a more generous limit, like 1 h, does not change results much). The table shows that the 15 concordance items in the experienced well-being measures using the HWB12 or Gallup response scales take <2 min on average. The same 15 items using the ELSA response scale takes more than 4 min. A seven-point response scale experienced question such as “Yesterday, did you feel happy” takes about 17 s to answer, while the same question asked on a five-point response scale (as in HWB12) takes about 8 s, and a binary response scale question (as in the Gallup questions) takes about 8 s as well. The evaluative measures (Cantril, Diener, SHARE and ONS) take very little time, not surprisingly. There is not much difference in duration across the waves.

**Table 1:** Duration in minutes of different well-being modules

Survey module	First wave	Second wave
HWB12 (15 items)	2.01	1.97
Gallup (15 items)	1.90	1.94
ELSA (15 items)	4.34	4.17
Cantril (Gallup)	1.24	1.13
SWLS	1.33	1.17
SHARE	0.20	0.19
ONS	0.86	0.81

Duration for respondents in minutes, restricted to sample with time lower or equal to 30 min for a module, or taking <90 s per experienced question

### Test–Retest Reliability of Measures

An important question of interest when fielding a survey on subjective well-being questions is the reliability of the resulting measures. We follow Krueger and Schkade (2008), and use a classical measurement error model $y_{i} = y_{i}^{*} + \epsilon_{i}$, where *y*_*i*_ is the observed well-being item measure, *y*_i_^*^ is the true value of the well-being item measure and $\epsilon_{i}$ is an error term assumed to have expectation zero. This set-up suggests a definition of the reliability ratio as the correlation coefficient of measures across waves $\left({r = corr\left({y_{i}^{1}, y_{i}^{2}} \right)} \right)$, where the superscripts refer to the waves in which the variables are measured. The reliability is thus measured here as a test–retest correlation between two waves of data, where the interval in our sample is at least 2 weeks and 26.6 days on average, as mentioned with respect to Fig. 1.

Table 2 shows the reliability ratios for all the evaluative subjective well-being measures. The Diener SWLS shows a reliability of about 0.80, which is very close to the estimate of 0.82 by Diener et al. (1985) who used an interval of 2 months, and the estimate by Alfonso et al. (1996) of 0.83, where the interval was 2 weeks between both measurements. As one would expect, the single item scales for evaluative well-being yield somewhat lower correlations, on the order of 0.67. The two ONS questions about yesterday are, as discussed earlier, really experienced measures, and we observe lower correlations amongst them between two waves. This reflects the fact that the specific reference to “yesterday” may pick up real changes in affect between different days. The Gallup measures referring to 5 years ago or 5 years in the future show lower reliability ratios than the one referring to the present, indicating possible error in recall of one’s situation 5 years ago and uncertainty about one’s future.

**Table 2:** Reliability ratios of the evaluative subjective well-being measures and ONS experienced measures (n = 3,938)

Satisfaction with life scale	*r*
In most ways, my life is close to ideal	0.68
The conditions of my life are excellent	0.72
I am satisfied with my life	0.73
So far I have gotten the important things I want in life	0.67
If I could live my life over, I would change almost nothing	0.65
SWLS^a^	0.79
SHARE	
How satisfied are you with your life in general?	0.67
Gallup	
On which step of the ladder would you say you stood 5 years ago?	0.59
On which step of the ladder would you say you stand now?	0.71
On which step of the ladder would you say you will stand on in the future, say about 5 years from now	0.66
ONS	
Overall, how satisfied are you with your life nowadays?	0.74
Overall, how happy did you feel yesterday?	0.57
Overall, how anxious did you feel yesterday?	0.45
Overall, to what extent do you feel that the things you do in your life are worthwhile?	0.65

^a^Computed as the average of the five satisfaction with life items

We also looked at correlations between the measures for experienced affect on the previous day presented in Table 3. As expected, we found lower correlations between waves, as changes may reflect both random measurement errors and true changes in the affect measures between the 2 days. Notice that the table shows correlations for all items, i.e. we include both the original items of each scale and the items added from the other scales. The correlations for those are underlined. Since we don’t use the ELSA limited DRM, but rather the ELSA response scale for all items that are in either Gallup or HWB12, all ELSA items are underlined. Recall that we did this so that we are able to compare response scale effects across a common set of items. Thus, a point of interest is to relate differences in correlations to differences in response scales (both the wording and the number of points on the response scale).

**Table 3:** Reliability ratios across waves of experienced subjective well-being measures

	ELSA	Gallup	HWB12
	n = 443	n = 477	n = 415
Happy	0.50	0.36	0.49
Interested	0.49	0.32	**0.42**
Content	0.40	0.39	0.54
Joyful	0.46	0.34	0.53
Enthusiastic	0.45	0.34	0.53
Frustrated	0.44	0.45	0.49
Sad	0.43	0.45	0.51
Angry	**0.33**	**0.28**	0.43
Tired	0.45	0.49	0.47
Stressed	0.43	0.41	0.50
Lonely	0.45	0.45	0.45
Worried	0.45	0.45	0.52
Bored	0.38	0.28	0.47
Pain	0.50	**0.49**	0.52
Depressed	**0.55**	0.41	**0.59**

Underlined correlations refer to items that have been added to the original scale; correlations in bold indicate the highest and lowest values in each column

The binary response scale used in the Gallup survey shows somewhat lower correlations across waves overall, with correlations between 0.28 and 0.49, in comparison with the five and seven point response scales used in the HWB-12 and ELSA questionnaires respectively. The ELSA response scale shows correlations ranging from 0.33 to 0.55, while the HWB12 response scale shows correlations between 0.42 and 0.59.

## The Relation Between Evaluative and Experienced Well-Being Measures

There is a lively debate in the literature on the dimensions of well-being and what different measures are capturing (for a review, see Diener 2000). Uniquely, our data bring together many of the currently used subjective well-being measures and thus allow us to investigate how they are related. To determine the relation between the various measures we conducted a number of different factor analyses.

As noted, we have all evaluative measures for all respondents, but each experienced measure is only available for a randomly chosen five ninth of the sample. In their original form, the Gallup and HWB12 measures are straightforward to use, since they produce ratings of a number of affect items. The ELSA questionnaire is more complicated to analyze as it asks for ratings for a number of activities during the previous day, so we use only the ELSA *response* *scale* for comparison with the response scales used by Gallup and in HWB12. In the current section the purpose is to consider the items in the original scales so we concentrate therefore initially on analyses of the Gallup and HWB12 measures. The ELSA response scale will be evaluated when studying the concordance items, which can be found in all three experienced well-being measures. Both analyses cover all evaluative measures as well as their respective experienced measures. We performed a factor analysis using principal components. In all cases factors are rotated orthogonally using the varimax method while we retain factors with eigenvalues greater than one.^5^

Table 4 presents the results for the Gallup case. The evaluative measures are grouped together in the upper part of the table and the Gallup experienced measures at the bottom. Factor loadings represent the direct effects of the factor on the observed variable (Bollen 1989). Large factor loadings (i.e. the largest number in absolute value on each row) are indicated in bold.

**Table 4:** Factor analysis: evaluative well-being and Gallup (original) experienced well-being (n = 2,724)

	Factor 1	Factor 2	Factor 3
Evaluative measures			
SWLS			
Ideal life	**0.8444**	−0.1733	0.1178
Excellent conditions	**0.8418**	−0.1836	0.1352
Satisfied	**0.8684**	−0.2143	0.1467
Important things	**0.7741**	−0.0999	0.1444
Change life	**0.7020**	−0.0984	0.0280
SHARE			
Satisfaction w life	**0.7953**	−0.2094	0.1600
ONS			
Satisfied nowadays	**0.8574**	−0.2373	0.1868
Happy	**0.6055**	−0.4860	0.3437
Anxious	−0.2000	0.0660	**−0.6268**
Worthwhile	**0.6754**	−0.3098	0.0896
Gallup			
Five years ago	**0.3736**	0.1720	0.2331
Now	**0.8461**	−0.2013	0.2029
Five years in future	**0.6494**	−0.2589	0.0018
Experienced measures			
Happy	−0.3308	**0.7785**	−0.1987
Interested	−0.1618	**0.5397**	0.0947
Joyful	−0.3114	**0.7738**	−0.1927
Sad	0.2862	−0.4429	**0.5342**
Angry	0.1257	−0.2470	**0.5678**
Stressed	0.1814	−0.2435	**0.6933**
Worried	0.2908	−0.2344	**0.6445**
Depressed	0.3211	−0.4114	**0.5497**
*Smile*	−0.2559	**0.7428**	−0.1166
*More days like this*	−0.2254	**0.6818**	−0.3656
*Treated w respect*	−0.1357	0.2443	**−0.4003**

The three items in italics are unique for Gallup and have not been included as items when we elicit responses using the HWB12 response scale of the Gallup response scale

Using the criterion of only retaining factors with eigenvalues greater than one,^6^ three factors are retained. The results confirm that evaluative and experienced well-being are distinct concepts. The evaluative measures form one factor, while the Gallup experienced measures appear to represent two factors. The factors representing experienced well-being form one positive and one negative affective dimension thus confirming that negative affect is not just the opposite of positive affect. This confirms prior findings of positive and negative affects as highly distinctive, orthogonal dimensions—not opposites that would be strongly negatively correlated—so that individuals can be experiencing both positive and negative affect simultaneously (Watson et al. 1988; Tuccitto et al. 2010). ONS-happy (“Overall, how happy did you feel yesterday?”) loads mainly on the evaluative first factor. Although the phrasing of the question would squarely put it in the experienced well-being domain, its location in the survey (right after an evaluative question, see “Appendix”) may have induced some respondents to use a global evaluation rather than focusing on yesterday’s affect.

Notably, ONS_worthwhile (“Overall, to what extent do you feel that the things you do in your life are **worthwhile**?”) does not appear to represent a different factor from the evaluative well-being factor. ONS-anxious loads on the negative affect factor, but with a surprising negative sign.

Table 5 shows the results when including the evaluative measures and the HWB12 experienced measures. In this case, four factors are retained, and their largest loadings in absolute value in each row are shown in bold. Again, the first factor represents evaluative well-being; the second factor now represents negative affect, while the third factor represents positive affect. The fourth factor mainly receives loadings from tired, bored, and pain, and thus represents a dimension related to fatigue rather than negative affect. These are all items that are not included in the Gallup item list. The items happy (“Yesterday, did you feel happy?”) and content (“Yesterday, did you feel content?”) load on all of the first three factors (negatively on the second, negative factor), while lonely (“Yesterday, did you feel lonely?”) loads negatively on factors 1 and 3, and positively on factors 2 and 4. ONS_happy (“Overall how happy did you feel yesterday”) loads on all of the first three factors, but negatively on the negative factor.

**Table 5:** Factor analysis: evaluative well-being and HWB12 (original) experienced well-being (n = 2,628)

	Factor 1	Factor 2	Factor 3	Factor 4
Evaluative measures				
SWLS				
Ideal life	**0.8304**	−0.1475	0.1430	−0.0544
Excellent conditions	**0.8393**	−0.1944	0.1240	−0.0532
Satisfied	**0.8552**	−0.1951	0.1778	−0.0568
Important things	**0.7725**	−0.1590	0.0626	−0.0553
Change life	**0.6817**	−0.1247	−0.0337	−0.0140
SHARE				
Satisfaction w life	**0.7783**	−0.1838	0.1801	−0.0562
ONS				
Satisfied nowadays	**0.8355**	−0.2175	0.1860	−0.1259
Happy	**0.5956**	−0.4473	0.3867	−0.1710
Anxious	−0.1491	**0.6386**	−0.0616	−0.0823
Worthwhile	**0.6770**	−0.0915	0.3178	−0.1114
Gallup				
Five years ago	**0.3842**	−0.0516	−0.2306	−0.2992
Now	**0.8348**	−0.2292	0.1262	−0.1790
Five years in future	**0.6392**	−0.0535	0.1827	−0.1073
Experienced measures				
Happy	0.4356	−0.4125	**0.6010**	−0.0487
Enthusiastic	0.3418	−0.2486	**0.6789**	−0.0116
Content	0.4718	−0.4061	**0.5352**	0.0034
Angry	−0.1516	**0.7107**	−0.1313	0.0817
Frustrated	0.1940	**0.7834**	−0.1757	0.1238
Tired	−0.1411	0.4244	−0.0695	**0.5566**
Sad	−0.2992	**0.6127**	−0.3349	0.2332
Stressed	−0.2085	**0.8307**	−0.1194	0.1244
Lonely	−0.3027	0.3154	**−0.4108**	0.3526
Worried	−0.2544	**0.7623**	−0.0984	0.1242
Bored	−0.1823	0.0542	−0.4818	**0.5596**
Pain	−0.1416	0.2527	0.0426	**0.6777**

Overall, a theme emerges of evaluative measures having different properties than experienced well-being measures. ONS Happy is somewhat of an exception, but as we observed before, the placement of this experienced well-being question immediately after an evaluative measures may have created confusion among respondents. We find that when conducting a factor analysis on both the Gallup and the HWB12 items, evaluative measures form a distinct factor from the experienced measures. We find differences in the number of dimensions for experienced measures, with a positive and negative factor in both Gallup and HWB12, as well as an additional fatigue factor for the HWB12 items. Our findings are in line with Headey et al. (1993), who conclude that life satisfaction and positive affect appear to measure relatively distinct dimensions (they find only a moderate correlation between the two dimensions). They also find that life satisfaction is highly negatively correlated with depression and moderately with anxiety and recommend measuring life satisfaction, positive affect, anxiety and depression separately so that we could better understand the causes and consequences of mental health.

There are two main differences between Gallup and HWB12: both the included items and the response scales differ. So without further analysis it is impossible to say if the added dimension is the result of added items or due to the response scale differences. In order to disentangle those two effects, the next section shows the results of factor analyses when including a set of common items, which only differ in the response scales used.

## The Effect of Response Scales

As noted in Sect. 2, we have added questions at the end of various experienced well-being modules to allow for crosswalks between different instruments. As a result of this, respondents who received the HWB12 module, the Gallup module, and the respondents who received the ELSA module answered the same items in number and nature, but with different response scales. The ELSA response scale is of the form: “Overall, how did you feel **yesterday**? Rate each feeling on a scale from 0—did not experience at all—to 6—the feeling was extremely strong”. The response scale in the HWB12 questionnaire is of the form (taking “happy” as an example): “Yesterday, did you feel **happy**? Would you say: not at all, a little, somewhat, quite a bit or very.” And finally, the Gallup question reads: “Did you experience **happiness** during a lot of the day yesterday? Yes or no”.

Thus, these items include both the original items of each scale and the items that were taken from the other scales. Tables 6, 7 and 8 therefore include all 15 experienced “concordance” measures—all with different response scales matching the original survey design.

**Table 6:** Factor analysis: experienced well-being, ELSA response scale (n = 2,703)

	ELSA 1	ELSA 2
	Troubled/fatigue	Positive
Happy	−0.3196	**0.8264**
Interested	−0.0976	**0.8245**
Frustrated	**0.8000**	−0.2594
Sad	**0.7917**	−0.2423
Enthusiastic	−0.1321	**0.8320**
Content	−0.2617	**0.7597**
Angry	**0.7605**	−0.1552
Tired	**0.6208**	−0.1525
Stressed	**0.7943**	−0.2286
Lonely	**0.6765**	−0.2002
Worried	**0.7841**	−0.1947
Bored	**0.5398**	−0.2488
Pain	**0.5700**	−0.0577
Depressed	**0.7845**	−0.3114
Joyful	−0.2053	**0.8429**

The largest loadings in absolute value in each row are shown in bold

**Table 7:** Factor analysis: experienced well-being, HWB12 response scale (n = 2,690)

	HWB12 1	HWB12 2	HWB12 3
	Troubled	Positive	Fatigue
Happy	−0.3960	**0.7557**	−0.1488
Interested	0.0111	**0.7319**	−0.1396
Frustrated	**0.8052**	−0.2309	0.1107
Sad	**0.6807**	−0.3042	0.3481
Enthusiastic	−0.1880	**0.8200**	−0.1060
Content	−0.3966	**0.7021**	−0.1197
Angry	**0.7607**	−0.1534	0.0468
Tired	0.3826	−0.1327	**0.5050**
Stressed	**0.8178**	−0.2460	0.1145
Lonely	0.3898	−0.2860	**0.5297**
Worried	**0.7726**	−0.1989	0.1610
Bored	0.0808	−0.2767	**0.7025**
Pain	0.2391	−0.0158	**0.6307**
Depressed	**0.6445**	−0.3208	0.4114
Joyful	−0.2603	**0.8241**	−0.1155

The largest loadings in absolute value in each row are shown in bold

**Table 8:** Factor analysis: experienced well-being, Gallup response scale (n = 2,788)

	Gallup 1	Gallup 2	Gallup 3
	Troubled	Positive	Fatigue
Happy	−0.3721	**0.7697**	−0.0493
Interested	0.1190	**0.6171**	−0.2177
Frustrated	**0.6671**	−0.2902	0.1720
Sad	**0.6635**	−0.3565	0.1717
Enthusiastic	−0.1024	**0.7373**	−0.1652
Content	−0.3562	**0.6537**	−0.0807
Angry	**0.6502**	−0.1301	0.0228
Tired	0.2744	−0.1293	**0.7053**
Stressed	**0.7055**	−0.1661	0.1593
Lonely	**0.4227**	−0.3293	0.2689
Worried	**0.6670**	−0.2073	0.2166
Bored	0.1814	−0.3684	**0.4283**
Pain	0.1250	−0.0655	**0.7736**
Depressed	**0.6716**	−0.3400	0.2205
Joyful	−0.3432	**0.7837**	−0.0504

The largest loadings in absolute value in each row are shown in bold

Table 6 displays the results of the factor analysis for experienced measures using the ELSA response scale. Two factors emerge when keeping factors with eigenvalues greater than one. The first factor, which we call “Troubled/Fatigue” represents negative affect, loading on frustration, sadness, anger, fatigue, stress, loneliness, worry, boredom, pain and depression. The second factor, which we simply call “Positive” groups the positive experienced measures, loading on happiness, interest, enthusiasm, content and joy.

We repeated this factor analysis using the HWB12 response scale (see Table 7). This time, three factors remained: a negative factor (factor #1, which we call “Troubled” mainly loading on: frustrated, sad, angry, stressed, worried, depressed), a positive factor (factor #2, which we call “Positive”, mainly loading on: happy, interested, enthusiastic, content, joyful), and a factor grouping items somewhat related to fatigue (factor #3, which we call “Fatigue”, mainly loading on: tired, lonely, bored, and pain).

Finally, when conducting the same analysis using the binary Gallup response scale, three factors remained (Table 8). The first (frustrated, sad, angry, lonely, worried, depressed, which we call “Troubled”) and third (tired, bored, pain, which we call “Fatigue”) are negative, while the second one (happy, interested, enthusiastic, content, joyful, which we call “Positive”) is positive. Note that three original items are dropped, asking whether the respondent smiled or laughed a lot, was treated with respect, or would wish to have more days just like yesterday.

A number of preliminary conclusions emerge. The number of factors retained is sensitive to the response scales used. The binary Gallup response scale yields three factors, the five-point HWB12 response scale yields three factors and the seven-point ELSA response scale yields two factors. This finding appears consistent with the older factor analysis literature where it has been observed that using categorical variables may lead to more factors, particularly if the distributions of the variables are skewed (see for example Lord and Novick 1968, or Olsson 1979). In comparison with Tables 4 and 5, where only original items were included, HWB12 yields the same number of experienced factors (3), but Gallup yielded two experienced and one evaluative factor when its original items were included, whereas with the common set of items the Gallup response scale yields three experienced factors. Thus, the fewer factors found in Table 4 are most likely due to the limited number of items included, as for instance boredom, fatigue, pain and loneliness are missing from the original Gallup response scale and indeed these contribute substantially to factor 3 in Table 8.

Factor analyses were also conducted on the common set of items, including evaluative measures (not shown here). The results in terms of the number of factors emerging remain quite similar, with one evaluative and two experienced factors when using the ELSA response scale, though it is worthwhile noticing that the ONS anxiety measure loads positively on the negative experienced factors rather than on the evaluative factor. HWB12 generates three experienced factors. In the Gallup case a fourth experienced factor (eigenvalue of 0.98) emerges representing mainly stress and pain. Interpreting the larger number of factors as an artefact of the cruder response scales suggests that it is advisable to use a response scale with a fairly large number of response categories, e.g. seven as in the ELSA response scale. In that case, experienced well-being can be described by two dimensions, one positive and one negative.

## Relation with Individual Characteristics

While an extensive literature exists on the determinants of evaluative well-being (see for example Dolan et al. 2008), much less is known of the determinants of experienced well-being. We concentrate here on demographic and socio-economic determinants. The motivation for this is that these appear most amenable to policy (e.g. with respect to income, work, education, or childcare), while there is a general interest in exploring how well-being varies with age, family composition (Deaton and Stone 2014) and gender. Furthermore it is of interest to explore to which extent determinants of evaluative well-being are different from those of experienced well-being and whether the different dimensions of experienced well-being have different determinants. We investigate how the well-being measures are related to demographic variables, including race, gender, education level, age bracket, having a partner, as well as socio-economic variables such as income bracket and working status, while we also include self-reported health and number of children in the household in our model. Our questionnaires also included questions about respondents’ major life events taken from the HRS, which will be analyzed in a separate paper.^7^ Formally, we specify the following model:

$$Y_{it}=\beta X_{it}+\epsilon_{it}$$

where *X*_*it*_ is a vector of covariates, while $\epsilon_{it}$ represents random error uncorrelated with the observable covariates. The subscript *t* indicates the wave (1 or 2) and *i* indexes the respondent. The model is estimated by ordinary least squares, where we allow for correlation of $\epsilon_{it}$ across the two waves (*t* = 1 or *t* = 1) by clustering standard errors on individuals.^8^ The simple equation specified here is not meant to provide a complete model of determinants of well-being and indeed one can imagine that causality sometimes runs from well-being to some of the right hand side variables. It is of interest nevertheless to investigate if the well-being measures covary with other variables in a plausible manner and to see if the relation between well-being and the right hand side variables is the same for each measure.

Table 9 shows the results for the evaluative measures. We have omitted the Gallup measures for 5 years ago and 5 years in the future; similarly for ONS we have only included the one true evaluative measure “Satisfied”. Given the different reference time frame used by those Gallup items and the experienced and eudemonic measures of the ONS scale, we chose to include only items referring to the present and involving evaluative measures. Looking at the effects of gender, we observe that these vary by outcome measure and are mostly insignificant. Men are less likely than women to agree with the statement “If I could live my life again, I would change almost nothing”. There currently is no consensus in the literature on the nature of differences in subjective well-being by sex, as some studies have shown higher levels of happiness for men (Haring et al. 1984) which could be related to higher prevalence of depression in women than men (Diener et al. 1999), while others have found that women report higher happiness (Alesina et al. 2004), and yet other studies have found no evidence of gender effects on subjective well-being (Louis and Zhao 2002; Dolan et al. 2008). Interactions between gender and education, income and having a partner did not yield any statistically significant results. Having a partner increases life satisfaction according to all measures. This result has also been found by others in the literature (see e.g. Dolan et al. 2008; Blanchflower and Oswald 2004). The presence of children in the household does not seem to consistently affect the well-being of the respondent, though as pointed out by Deaton and Stone (2013), this could be a function of controlling for factors associated with having children, such as being married, richer, and healthier. The results also show that by and large Blacks and Hispanics report higher subjective well-being than non-Hispanic Whites. Concerning education, the reference category for the education variables is “graduate education”. Although many coefficients are not statistically significantly different from zero, all significant coefficients confirm Oswald and Blanchflower’s finding of a positive relationship between education and well-being (2004).

**Table 9:** Regression of evaluative well-being measure on demographic and socioeconomic status variables

	Gallup	SWLS items					SWLS		ONS	SHARE
		Ideal life	Excellent cond.	Satisfied	Important things	Change life	Factor	Average	Satisfied	Satisfied
Male	0.00202 (0.0674)	−0.0161 (0.0610)	0.0409 (0.0599)	−0.00260 (0.0613)	−0.126** (0.0583)	−0.211*** (0.0716)	−0.0332 (0.0345)	−0.0612 (0.0537)	−0.0002 (0.0745)	−0.00910 (0.0266)
With partner	0.459*** (0.0805)	0.325*** (0.0718)	0.313*** (0.0695)	0.407*** (0.0715)	0.588*** (0.0696)	0.441*** (0.0801)	0.268*** (0.0403)	0.420*** (0.0625)	0.526*** (0.0881)	0.191*** (0.0313)
Other	−0.195 (0.199)	−0.378** (0.182)	−0.339** (0.171)	−0.358** (0.182)	−0.313* (0.165)	0.0516 (0.182)	−0.185* (0.100)	−0.269* (0.155)	−0.271 (0.217)	−0.214*** (0.0769)
Black	0.342*** (0.124)	0.182* (0.105)	0.0891 (0.104)	0.228** (0.107)	−0.102 (0.104)	0.0837 (0.116)	0.0648 (0.0567)	0.0979 (0.0878)	0.489*** (0.130)	0.123*** (0.0467)
Hispanic	0.327** (0.130)	0.267** (0.106)	0.309*** (0.111)	0.223** (0.112)	0.144 (0.110)	0.206* (0.120)	0.140** (0.0630)	0.215** (0.0972)	0.362** (0.145)	0.0754 (0.0493)
No HS	0.160 (0.226)	0.115 (0.183)	−0.0283 (0.186)	0.0275 (0.203)	−0.278 (0.190)	−0.0423 (0.213)	−0.0369 (0.107)	−0.0617 (0.167)	0.0740 (0.245)	0.118 (0.0834)
HS degree	−0.145 (0.120)	−0.0262 (0.111)	−0.210* (0.110)	−0.111 (0.110)	−0.321*** (0.104)	−0.0617 (0.128)	−0.0953 (0.0630)	−0.145 (0.0978)	−0.191 (0.135)	−0.0392 (0.0473)
Some college	−0.102 (0.0893)	−0.0226 (0.0847)	−0.183** (0.0837)	−0.104 (0.0843)	−0.241*** (0.0773)	−0.0505 (0.103)	−0.0783 (0.0478)	−0.119 (0.0744)	−0.0597 (0.100)	−0.0343 (0.0363)
Bachelor	−0.0859 (0.0899)	−0.0124 (0.0868)	−0.0748 (0.0842)	−0.0693 (0.0851)	−0.133* (0.0767)	−0.0207 (0.106)	−0.0420 (0.0481)	−0.0636 (0.0750)	−0.0320 (0.100)	−0.0123 (0.0370)
<$25,000$	−0.650*** (0.141)	−0.768*** (0.126)	−0.884*** (0.123)	−0.731*** (0.126)	−0.740*** (0.118)	−0.572*** (0.142)	−0.477***	−0.732*** (0.110)	−0.785*** (0.155)	−0.216*** (0.0555)
							(0.0710)			
$25,000–$49,999	−0.388*** (0.102)	−0.596*** (0.0950)	−0.676*** (0.0945)	−0.561*** (0.0921)	−0.558*** (0.0883)	−0.420*** (0.115)	−0.367*** (0.0535)	−0.563*** (0.0833)	−0.456*** (0.111)	−0.182*** (0.0417)
$50,000–$74,999	−0.298*** (0.0905)	−0.370*** (0.0866)	−0.459*** (0.0868)	−0.365*** (0.0842)	−0.385*** (0.0793)	−0.373*** (0.108)	−0.251*** (0.0485)	−0.389*** (0.0755)	−0.301*** (0.0999)	−0.109*** (0.0372)
$75,000–$100,000	−0.00398 (0.104)	−0.0723 (0.0992)	−0.134 (0.0972)	−0.0628 (0.0940)	−0.0995 (0.0879)	−0.0726 (0.123)	−0.0563 (0.0560)	−0.0867 (0.0875)	−0.0469 (0.116)	−0.0246 (0.0416)
Age <25	0.0833 (0.251)	0.316 (0.201)	0.516** (0.206)	0.431** (0.215)	0.0565 (0.205)	0.573** (0.253)	0.242* (0.124)	0.384** (0.193)	0.454* (0.269)	0.135 (0.0991)
Age 25–35	−0.322** (0.136)	0.0654 (0.123)	0.153 (0.123)	0.0597 (0.119)	−0.239** (0.119)	0.315** (0.145)	0.0381 (0.0704)	0.0703 (0.109)	−0.173 (0.146)	0.00338 (0.0527)
Age 35–45	−0.430*** (0.137)	0.0117 (0.130)	0.00148 (0.130)	−0.0927 (0.124)	−0.316*** (0.123)	−0.0764 (0.151)	−0.0575 (0.0734)	−0.0909 (0.114)	−0.323** (0.154)	−0.0847 (0.0542)
Age 45–55	−0.567*** (0.131)	−0.381*** (0.122)	−0.352*** (0.122)	−0.397*** (0.120)	−0.480*** (0.114)	−0.424*** (0.138)	−0.260*** (0.0690)	−0.407*** (0.107)	−0.563*** (0.146)	−0.196*** (0.0517)
Age 55–65	−0.292*** (0.108)	−0.0423 (0.102)	−0.0898 (0.103)	−0.0663 (0.0977)	−0.157* (0.0927)	−0.280** (0.124)	−0.0739 (0.0578)	−0.125 (0.0900)	−0.289** (0.120)	−0.0830* (0.0437)
Unemployed	−0.611*** (0.185)	−0.374** (0.145)	−0.430*** (0.141)	−0.485*** (0.159)	−0.318** (0.153)	−0.128 (0.161)	−0.232*** (0.0858)	−0.348*** (0.133)	−0.696*** (0.198)	−0.210*** (0.0704)
Retired	0.619*** (0.109)	0.549*** (0.103)	0.503*** (0.104)	0.591*** (0.0979)	0.483*** (0.0918)	0.481*** (0.128)	0.338*** (0.0583)	0.522*** (0.0910)	0.687*** (0.121)	0.201*** (0.0440)
Disabled	−0.462** (0.209)	−0.377** (0.171)	−0.479*** (0.161)	−0.476*** (0.184)	−0.364** (0.179)	−0.112 (0.177)	−0.244** (0.0982)	−0.364** (0.152)	−0.394* (0.224)	−0.183** (0.0781)
Other work	0.164 (0.101)	0.231** (0.0911)	0.235*** (0.0909)	0.217** (0.0920)	0.157* (0.0881)	0.215** (0.106)	0.137*** (0.0520)	0.213*** (0.0807)	0.218* (0.113)	0.0569 (0.0405)
Self-reported health	−0.701*** (0.0456)	−0.561*** (0.0389)	−0.642*** (0.0371)	−0.583*** (0.0386)	−0.375*** (0.0368)	−0.455*** (0.0420)	−0.340*** (0.0218)	−0.524*** (0.0338)	−0.780*** (0.0488)	−0.264*** (0.0169)
Children in HH	−0.0782 (0.0737)	−0.0840 (0.0665)	−0.157** (0.0663)	−0.0900 (0.0669)	0.111* (0.0650)	−0.0127 (0.0763)	−0.0316 (0.0380)	−0.0451 (0.0590)	−0.0663 (0.0811)	−0.00881 (0.0288)
Constant	9.043*** (0.167)	6.334*** (0.152)	6.637*** (0.152)	6.711*** (0.144)	6.491*** (0.143)	5.288*** (0.182)	1.071*** (0.0855)	6.286*** (0.133)	8.975*** (0.182)	3.837*** (0.0640)
Observations	4,996	4,997	4,990	4,992	4,993	4,993	4,978	4,978	4,997	4,995
R^2^	0.216	0.193	0.246	0.219	0.196	0.116	0.249	0.246	0.225	0.196
*p* value race	0.00	0.00	0.00	0.01	0.08	0.37	0.02	0.02	0.00	0.00
*p* value education	0.50	0.94	0.15	0.72	0.01	0.99	0.49	0.51	0.63	0.28
*p* value income	0.00	0.00	0.00	0.00	0.00	0.00	0.00	0.00	0.00	0.00
*p* value age	0.00	0.00	0.00	0.00	0.00	0.00	0.00	0.00	0.00	0.00
*p* value work status	0.00	0.00	0.00	0.00	0.00	0.00	0.00	0.00	0.00	0.00

Estimation method is OLS; standard errors are clustered at the individual level. The *p* values mentioned in the last rows refer to a test of joint significance of the indicator variables for the categories race, education, income, age, and work status

Subjective well-being increases monotonically with income according to all evaluative measures. In comparison to the reference category of respondents reporting an income above $100,000, we observe large negative and statistically significant coefficients for most lower income groups. The size of those coefficients suggests an almost linear relationship between income and subjective well-being measures in this income range. A positive relation between income and subjective well-being has been found many times in the literature, with existing research suggesting positive but diminishing returns to income (Dolan et al. 2008).

The reference category for age consists of respondents over 65. Several studies have suggested a “U-shape” in age with the lowest life satisfaction occurring in middle age (Dolan et al. 2008; Blanchflower and Oswald 2004). By and large that pattern is confirmed for the various well-being measures in the table. We observe that self-reported health—here coded as 1 being Excellent, and 5 being Poor so that a negative sign represents a higher level of health—is strongly correlated with well-being, which corresponds to general findings in the literature (Diener et al. 1999; Helliwell 2003).

With regards to working status, we used the category “working now” as a reference group, so that the results for individuals who are retired, disabled, unemployed, or in a different working situation (homemakers, or on sick leave, temporarily laid-off or other) represent differences with “working now”. Consistent with the literature, we observe a strong negative effect of being unemployed (see for instance Clark and Oswald 1994; Stutzer 2004; or DiTella et al. 2001). We also find a negative effect for being disabled, which appears in line with studies challenging the theory of hedonic adaptation whereby individuals suffering major changes in life circumstances, such as the onset of a disability, return to baseline levels of happiness (Lucas 2007). We also confirm prior findings (Kim and Moen 2002) of a strong positive relation between being retired and subjective well-being. Being in “Other work” has a positive, though not always significant, effect on subjective well-being.

Finally, the last five rows show the *p* values of joint significance tests for each category of characteristics. We cannot reject the hypothesis of no difference between the education categories except for the question “So far, I have gotten the important things I want in life”. Virtually all other categories are jointly significant.

The coefficients in Table 9 are not directly comparable across columns as the dependent variables are measured on different scales. However if the scales would be the only difference between the dependent variables, then coefficients in different columns should be fixed multiples of each other.^9^ Table 10 summarizes the results of tests of proportionality of coefficients across the various models in Table 9. The Null Hypothesis for all the tests is formulated as follows: $H_{0} = \frac{{\beta_{1,\,model\,1}}}{{\beta_{1,\,model\,2}}} = \frac{{\beta_{2,\,model\,1}}}{{\beta_{2,\,model\,2}}},$ etc. The entries in the table are the *p* values of tests of the null hypothesis for each of the pairs of models that we are considering. We observe that out of all ten possible combinations, the Null Hypothesis of proportionality of coefficients gets rejected at the 5 % level four times. All four rejections involve either the SWLS based on averaging the item scores or the SWLS based on factor analysis.^10^ Inspecting the five items that constitute the SWLS makes it clear that only one item (“I am satisfied with my life”) corresponds with the simple one shot questions of SHARE, ONS, and Gallup. This suggests that the SWLS measures a somewhat broader concept of evaluative well-being than the other three measures. Yet, remarkably in the factor analyses presented earlier, it appeared that the items on the SWLS all loaded on the same factor along with the SHARE, ONS, and Gallup items on an overall evaluative dimension.

**Table 10:** Testing the proportionality of coefficients—evaluative measures (*p* values)

	Gallup now	SWLS factor	SWLS average	ONS satisfaction
SWLS factor	0.01			
SWLS average	0.01	0.09		
ONS satisfaction	0.89	0.02	0.02	
SHARE satisfaction	0.52	0.35	0.32	0.67

The null hypothesis tested here is $H_{0} = \frac{{\beta_{1,\,model\,1}}}{{\beta_{1,\,model\,2}}} = \frac{{\beta_{2,\,model\,1}}}{{\beta_{2,\,model\,2}}},$ etc. therefore testing the proportionality of coefficients across pairs of models. The table shows *p* values of the test statistics corresponding to the null hypothesis for each pair of models

Table 11 shows the results of regressions for the explanation of experienced well-being measures. The dependent variables are scales based on factor loadings from factor analyses presented in Tables 6, 7 and 8. So in all cases the scales are based on the common set of items. It is of interest to not only compare the scales (which are only different because of differences in response scales), but also between the experienced scales and the evaluative scales, for which regressions were presented in Table 9. For both the ELSA and HWB12 scales males score lower on the negative affect (“Troubled”) scale (but marginally significantly positive for the Gallup scale). Here again, interactions between gender and education, income and having a partner did not yield any statistically significant results. Having a partner has little effect on experienced well-being (although the HWB12 scale suggests a somewhat lower score on the “Fatigue” scale), in contrast to the findings for the evaluative well-being scales where the presence of a partner has a strong positive effect.

**Table 11:** Regression of experienced scales on demographic and socioeconomic status variables

	ELSA		Gallup			HWB12		
	Troubled/fatigue	Positive	Troubled	Positive	Fatigue	Troubled	Positive	Fatigue
Male	−0.166*** (0.0511)	−0.0536 (0.0539)	0.0857* (0.0497)	0.0157 (0.0550)	−0.000920 (0.0509)	−0.142** (0.0551)	−0.0469 (0.0566)	0.0222 (0.0501)
With partner	−0.0248 (0.0603)	0.0706 (0.0626)	0.0581 (0.0566)	−0.140** (0.0628)	−0.0458 (0.0581)	0.0235 (0.0629)	0.139** (0.0659)	−0.166*** (0.0598)
Other	−0.0499 (0.144)	0.0261 (0.153)	−0.0425 (0.134)	0.130 (0.158)	−0.0525 (0.142)	−0.175 (0.132)	−0.141 (0.185)	0.0384 (0.140)
Black	−0.171 (0.105)	0.219** (0.0898)	0.201** (0.0827)	−0.176* (0.0987)	0.197** (0.0862)	−0.153 (0.120)	−0.0865 (0.106)	−0.0873 (0.105)
Hispanic	0.0704 (0.0927)	0.318*** (0.0866)	−0.376*** (0.106)	−0.250** (0.0995)	0.0727 (0.0994)	0.112 (0.100)	0.133 (0.0934)	0.221** (0.111)
No HS	−0.0590 (0.196)	−0.242 (0.184)	0.134 (0.197)	0.277 (0.193)	−0.110 (0.160)	−0.379** (0.165)	−0.282* (0.163)	0.480** (0.193)
HS degree	−0.143 (0.0875)	−0.0870 (0.0931)	0.0197 (0.0871)	0.273*** (0.0933)	−0.118 (0.0912)	−0.407*** (0.0942)	−0.264*** (0.0973)	0.159* (0.0897)
Some college	−0.0229 (0.0679)	0.0941 (0.0752)	0.0379 (0.0648)	0.105 (0.0744)	−0.0713 (0.0676)	−0.227*** (0.0819)	−0.188** (0.0809)	0.102 (0.0716)
Bachelor	−0.0933 (0.0651)	0.0506 (0.0720)	−0.0138 (0.0698)	0.0449 (0.0790)	−0.0267 (0.0731)	−0.125 (0.0828)	−0.0440 (0.0806)	−0.0593 (0.0741)
<$25,000$	0.0956 (0.0974)	−0.0297 (0.104)	−0.0936 (0.0998)	0.165 (0.115)	−0.252** (0.100)	0.156 (0.105)	0.0799 (0.113)	−0.0642 (0.101)
$25,000–$49,999	0.00674 (0.0767)	−0.00399 (0.0799)	0.0145 (0.0767)	−0.0194 (0.0837)	−0.262*** (0.0791)	0.101 (0.0814)	0.0491 (0.0909)	−0.0853 (0.0797)
$50,000–$74,999	0.0210 (0.0674)	−0.0421 (0.0737)	−0.0457 (0.0732)	0.125 (0.0832)	−0.157** (0.0720)	0.0443 (0.0786)	0.0698 (0.0817)	−0.0416 (0.0737)
$75,000–$100,000	−0.00352 (0.0784)	0.0149 (0.0822)	−0.00880 (0.0754)	−0.0384 (0.0870)	−0.0969 (0.0816)	−0.0275 (0.0879)	0.130 (0.0915)	−0.0476 (0.0765)
Age <25	0.295* (0.176)	−0.154 (0.202)	−0.500*** (0.190)	−0.0891 (0.217)	−0.155 (0.229)	0.0147 (0.188)	−0.265 (0.208)	0.166 (0.201)
Age 25–35	0.305*** (0.0978)	0.0244 (0.116)	−0.180* (0.0989)	0.186* (0.101)	−0.101 (0.0979)	0.280** (0.120)	−0.185 (0.123)	0.310*** (0.105)
Age 35–45	0.216** (0.0997)	−0.0839 (0.121)	−0.234** (0.0965)	0.186* (0.103)	0.00833 (0.0959)	0.236** (0.112)	−0.260** (0.123)	0.0214 (0.100)
Age 45–55	0.307*** (0.0922)	−0.113 (0.111)	−0.0960 (0.0890)	0.208** (0.0960)	−0.108 (0.0887)	0.215** (0.105)	−0.395*** (0.110)	0.0106 (0.0932)
Age 55–65	0.177** (0.0739)	0.0636 (0.0986)	−0.163** (0.0754)	0.138* (0.0833)	−0.00543 (0.0806)	0.0615 (0.0898)	−0.178* (0.1000)	0.102 (0.0808)
Unemployed	−0.0624 (0.127)	−0.0494 (0.123)	−0.210 (0.131)	0.202 (0.152)	−0.0793 (0.119)	0.219 (0.141)	−0.0966 (0.126)	0.171 (0.120)
Retired	−0.186** (0.0737)	0.248** (0.1000)	0.188** (0.0766)	0.0372 (0.0861)	−0.0907 (0.0839)	−0.233** (0.0954)	0.0123 (0.101)	0.105 (0.0836)
Disabled	0.264 (0.172)	−0.0649 (0.131)	−0.196 (0.151)	−0.0228 (0.137)	−0.370*** (0.130)	0.0720 (0.157)	−0.0919 (0.141)	0.715*** (0.151)
Other work	−0.0482 (0.0831)	0.119 (0.0799)	0.00621 (0.0714)	−0.0871 (0.0750)	−0.00191 (0.0713)	0.00677 (0.0772)	0.0753 (0.0868)	0.00651 (0.0856)
Self-reported health	0.239*** (0.0302)	−0.200*** (0.0340)	−0.148*** (0.0310)	0.183*** (0.0314)	−0.280*** (0.0305)	0.167*** (0.0341)	−0.182*** (0.0338)	0.167*** (0.0341)
Children in HH	0.0651 (0.0551)	0.0670 (0.0571)	−0.00575 (0.0546)	0.0851 (0.0582)	0.00544 (0.0538)	0.0643 (0.0585)	0.0779 (0.0601)	−0.0228 (0.0535)
Constant	−0.709*** (0.116)	0.349*** (0.131)	0.486*** (0.119)	−0.659*** (0.129)	1.079*** (0.116)	−0.404*** (0.135)	0.643*** (0.147)	−0.566*** (0.132)
Observations	1,678	1,678	1,683	1,683	1,683	1,596	1,596	1,596
R^2^	0.115	0.077	0.077	0.067	0.128	0.081	0.073	0.119
*p* value race	0.30	0.00	0.00	0.02	0.13	0.17	0.30	0.17
*p* value education	0.35	0.07	0.90	0.04	0.71	0.00	0.02	0.01
*p* value income	0.86	0.96	0.75	0.08	0.02	0.42	0.71	0.89
*p* value age	0.02	0.15	0.03	0.25	0.47	0.07	0.01	0.01
*p* value work status	0.04	0.06	0.02	0.39	0.05	0.03	0.72	0.00

Estimation method is OLS; standard errors are clustered at the individual level. The *p* values mentioned in the last rows refer to a test of joint significance of the indicator variables for the categories race, education, income, age, and work status

The effect of ethnicity is hard to summarize. According to the ELSA scale Hispanics and Blacks experience more positive affect compared to whites and non-Hispanic whites. According to the Gallup scales Blacks and Hispanics experience less positive affect, while the HWB12 scale shows no significant effects of ethnicity on positive affect. For Blacks we find more negative affect for the Gallup scale. Hispanics are less troubled according to the Gallup scale and more tired according to the HWB12 scale. Education also shows patterns that vary by response scale. The ELSA and Gallup scales show few significant effects. The HWB12 scale suggests that individuals with lower education experience less positive affect, while they are also less troubled, but more tired, bored and suffering from pain.

The most striking contrast between evaluative and experienced well-being is the effect of income. Whereas for evaluative well-being we observe a strong positive relation with income, such a relation is hardly discernible for experienced well-being. This result is somewhat stronger than earlier findings by Kahneman and Deaton (2010), who found that while life evaluation items rise steadily with socio-economic status, experienced measures of well-being do not improve beyond an annual income of approximately $75,000. Here we find very little evidence of a relation with income, although interestingly the Gallup scale produces marginally significant effects, which also is the scale used by Kahneman and Deaton (2010). Similarly, we observe that the U-shaped relation with age that we observed for evaluative well-being does not show up for experienced well-being. The results for labor market status show few consistent patterns across scales. As with evaluative well-being, health is an important determinant of experienced well-being. Both the ELSA and the HWB12 scale show that better health is associated with more positive affect and less negative affect (remember that Health is coded 1–5, so that a higher number means less good health). However for the Gallup scale the effects are reversed.

Joint tests of significance for each category of respondent characteristics do not reject the null of no effect for education (with the exception of the HWB12 factors), income, age (with the exception ELSA “Troubled/Fatigue” scale and the HWB12 factors), and race (with the exception of ELSA “Positive” and Gallup “Troubled” and “Positive”). Work status shows the strongest effects. Only Gallup “Positive” and HWB12 “Positive” do not show a significant relation.

Table 12 presents results of proportionality tests of coefficients in the various columns of Table 12, analogous to the results presented in Table 10. Since the positive and negative affect scales are assumed to tap different dimensions, we would not expect the proportionality hypothesis to hold for the different affect scales within ELSA, Gallup, and HWB12. For ELSA and HWB12 that is indeed the case, *p* values are 0.02 and 0.04 respectively. For Gallup this does not seem to be the case however: the null of proportionality between the three different affect scales does not get rejected. A second relation of interest is to see if the positive affect scales across ELSA, Gallup, and HWB12 satisfy proportionality. That indeed is confirmed by the entries in the table; *p* values are 0.77, 0.59, and 0.92. Thirdly we consider the negative affect scales. Here the expected patterns are somewhat less clear-cut as the negative affect scales vary somewhat across ELSA, Gallup, and HWB12. We do observe that the null of proportionality between ELSA Troubled/Fatigue and the Gallup and HWB12 Troubled and Fatigue scales gets easily accepted. Similarly we cannot reject the null of proportionality between HWB12 Troubled and Gallup Troubled, and between HWB12 Fatigue and Gallup Fatigue. On the other hand, HWB12 Troubled and Gallup Fatigue do not pass the null of proportionality, indeed suggesting that these scales measure something different.

**Table 12:** Testing the proportionality of coefficients—experienced measures (*p* values)

	ELSA		Gallup			HWB12	
	Troubled/fatigue	Positive	Troubled	Positive	Fatigue	Troubled	Positive
ELSA positive	0.02						
Gallup troubled	0.47	0.04					
Gallup positive	0.20	0.77	0.88				
Gallup fatigue	0.85	0.97	0.96	0.99			
HWB12 troubled	0.43	0.02	0.66	0.04	0.01		
HWB12 positive	0.16	0.59	0.79	0.92	0.22	0.04	
HWB12 fatigue	0.19	0.33	0.82	0.67	0.09	0.19	0.89

The null hypothesis tested here is $H_{0} = \frac{{\beta_{1,\,model\,1}}}{{\beta_{1,\,model\,2}}} = \frac{{\beta_{2,\,model\,1}}}{{\beta_{2,\,model\,2}}},$ etc. therefore testing the proportionality of coefficients across pairs of models. The table shows *p* values of the test statistics corresponding to the null hypothesis for each pair of models

## Conclusions

It is increasingly understood that traditional economic measures are necessary, but not sufficient, to measure societal progress (Stiglitz et al. 2009). Accordingly, in recent decades, research interest has been rising to find broader measures of well-being to be used to monitor societal progress and evaluate policy. The literature thus far has conceptualized subjective well-being either as the evaluation of life satisfaction/dissatisfaction (evaluative well-being measures) or as the combination of experienced affect (range of emotions from joy to misery).

In this paper, we conducted an experiment to investigate the relations between a number of evaluative and experienced measures (and one eudemonic measure), using the ALP. This is the first time that all these different types of measures have been collected jointly in a population survey. Although the concepts asked in the different experienced measures included in our experiment are in some cases the same, measures differ in the scales of their questions and so, we also studied the correspondence across these different scales. The experiment confirms a number of findings in the literature and yields some new results.

We find that all evaluative measures load on the same factor. Although this would suggest that there is not much to choose among them, the test results presented in Table 10, show that the SWLSs (both the one based on averaging items and the one based on factor analysis) have a different relation with demographics and self-reported health than the other three single item scales. Hence, for analyses of determinants of subjective well-being it does matter which measure one uses. The ONS-flourishing (eudemonic) measure does not seem to represent a separate factor; it mainly loads on the common evaluative factor.

The positive and negative experienced affect measures load on different factors, thus confirming that positive and negative affect are not simply opposite poles on the same scale. Depending on the response scale used, we find that negative affect can be represented by one or two factors. The ONS-happy measure loads both on the evaluative factor and on both the positive and negative affect factor. It is not entirely clear why this happens, but one possibility is the design of the ONS questionnaire, which places this experienced measure directly behind an evaluative question. Both previous points suggest the need for more work on the structure of questionnaires (response scales, lay-out, question order, etc.).

The relation of evaluative and experienced measures with demographics is markedly different. For instance, evaluative well-being increases monotonically and almost linearly with income; for experienced well-being no such relation with income is found. Evaluative well-being shows a U-shaped relation with age, while for experienced well-being no such relation is found. Also, health and labor market status, which have clear and significant effects on evaluative well-being, do not appear to have much of a consistent influence on experienced well-being. Whether one finds a relation or not appears to depend on the kind of response scale used in eliciting items. In general, it appears that the relation between experienced measures and demographics is much weaker than between evaluative measures and demographics.

The paper pays a fair bit of attention to the effect of response scales used for the affect measures. The different response scales imply a different number of underlying factors and different relations with demographics. This is clearly undesirable given that they all are based on the same items. The relation between experienced well-being and personal circumstances and demographics should not depend on whether we use a binary response scale, a five-point response scale, or a seven-point response scale. In a number of ways the ELSA seven-point response scale appears to behave better than the other coarser response scales (especially the Gallup response scales). This result confirms the theory of higher data quality, through higher validity and lower residual error, when using a higher number of answer categories (Andrews 1984). Partly this can be ascribed to the fact that with finer response scales, respondents can express their feelings in a more nuanced way, while assumptions of underlying normal distributions (which motivate many of the statistical procedures) will be closer to being satisfied by the data.

## Appendix: Questionnaires

### Evaluative Questions

#### The Cantril Ladder: Gallup Well-Being Index

Please imagine a ladder with steps numbered from 0 at the bottom to 10 at the top. Suppose we say that the top of the ladder represents the best possible life for you and the bottom of the ladder represents the worst possible life for you. On which step of the ladder would you say you personally feel you stand at this time, assuming that the higher the step the better you feel about your life, and the lower the step the worse you feel about it? **Which step comes closest to the way you feel**? *1/2/3/4/5/6/7/8/9/10*

Please imagine a ladder with steps numbered from 0 at the bottom to 10 at the top. Suppose we say that the top of the ladder represents the best possible life for you and the bottom of the ladder represents the worst possible life for you. On which step of the ladder would you say you stood **5 years ago**? *1/2/3/4/5/6/7/8/9/10*

Please imagine a ladder with steps numbered from 0 at the bottom to 10 at the top. Suppose we say that the top of the ladder represents the best possible life for you and the bottom of the ladder represents the worst possible life for you. On which step of the ladder would you say you will stand on in the future, say about **5** **years from now**? *1/2/3/4/5/6/7/8/9/10*

#### Diener’s Life Satisfaction: HRS/ELSA

Please say how much you agree or disagree with the following statements.

- **In most ways my life is close to ideal**. *Strongly disagree/Somewhat disagree/Slightly disagree/Neither agree or disagree/Slightly agree/Somewhat agree/Strongly agree*
- **The conditions of my life are excellent**. *Strongly disagree/Somewhat disagree/Slightly disagree/Neither agree or disagree/Slightly agree/Somewhat agree/Strongly agree*
- **I am satisfied with my life**. *Strongly disagree/Somewhat disagree/Slightly disagree/Neither agree or disagree/Slightly agree/Somewhat agree/Strongly agree*
- **So far, I have gotten the important things I want in life**. *Strongly disagree/Somewhat disagree/Slightly disagree/Neither agree or disagree/Slightly agree/Somewhat agree/Strongly agree*
- **If I could live my life again, I would change almost nothing**. *Strongly disagree/Somewhat disagree/Slightly disagree/Neither agree or disagree/Slightly agree/Somewhat agree/Strongly agree*

#### Life Satisfaction: SHARE

How satisfied are you with your life in general? *Very satisfied/Somewhat satisfied/Somewhat dissatisfied/Very dissatisfied*

#### ONS: ELSA

- Overall, how **satisfied** are you with your life nowadays? *(Not at all) 0/1/2/3/4/5/6/7/8/9/10 (Completely)*
- Overall, how **happy** did you feel yesterday? *(Not at all) 0/1/2/3/4/5/6/7/8/9/10 (Completely)*
- Overall, how **anxious** did you feel yesterday? *(Not at all) 0/1/2/3/4/5/6/7/8/9/10 (Completely)*
- Overall, to what extent do you feel that the things you do in your life are **worthwhile**? *(Not at all) 0/1/2/3/4/5/6/7/8/9/10 (Completely)*

### Experienced Questions: ELSA

Now, please pause briefly to think about **yesterday**, from the morning until the end of the day. Think about where you were, what you were doing, who you were with, and how you felt.

- What day of the week was it **yesterday**?
- What time did you wake up **yesterday**?
- What time did you go to sleep at the end of the day **yesterday**?
- Yesterday, did you feel any **pain**? *None/A little/Some/Quite a bit/A lot*
- Did you feel well-rested **yesterday morning** (that is, you slept well the night before)? *Yes/No*
- Was **yesterday** a normal day for you or did something unusual happen? *Yes, just a normal day/No, my day included unusual bad (stressful) things/No, my day included unusual good things*

Please think about the **things you did yesterday**. How did you spend your time and how did you feel?

- Yesterday, did you **watch TV**? *Yes/No* (skip next 2 question)
- How much time did you spend **watching TV yesterday**?
- How did you feel when you were **watching TV yesterday**?
  - Matrix showing: *Happy/Interested/Frustrated/Sad*
- Yesterday, did you **work or volunteer**? *Yes/No* (skip next 2 question)
- How much time did you spend **working or volunteering yesterday**?
- How did you feel when you were **working or volunteering yesterday**? Rate each feeling on a scale from 0—did not experience at all—to 6—the feeling was extremely strong.
  - Matrix showing: *Happy/Interested/Frustrated/Sad*
- Yesterday, did you go for a **walk or exercise**? *Yes/No* (skip next 2 question)
- How much time did you spend **walking or exercising yesterday**?
- How did you feel when you were **walking or exercising yesterday**? Rate each feeling on a scale from 0—did not experience at all—to 6—the feeling was extremely strong.
  - Matrix showing: *Happy/Interested/Frustrated/Sad*
- Yesterday did you do any **health-related activities other than walking or exercise**? For example, visiting a doctor, taking medications or doing treatments. *Yes/No* (skip next 2 question)
- How much time did you spend doing **health-related activities yesterday**?
- How did you feel when you were **doing health-related activities yesterday**? Rate each feeling on a scale from 0—did not experience at all—to 6—the feeling was extremely strong.
  - Matrix showing: *Happy/Interested/Frustrated/Sad*
- Yesterday did you **travel or commute**? E.g. by car, train, bus etc. *Yes/No* (skip next 2 question)
- How much time did spend **travelling or commuting yesterday?**
- How did you feel when you were **travelling or commuting yesterday**? Rate each feeling on a scale from 0—did not experience at all—to 6—the feeling was extremely strong.
  - Matrix showing: *Happy/Interested/Frustrated/Sad*
- Yesterday did you **spend** **time with friends or family**? *Yes/No* (skip next 2 question)
- How much time did you spend **with friends or family yesterday?**
- How did you feel when you were **with friends or family yesterday**? Rate each feeling on a scale from 0—did not experience at all—to 6—the feeling was extremely strong.
  - Matrix showing: *Happy/Interested/Frustrated/Sad*
- Yesterday did you **spend time at home by yourself**? Without a spouse, partner or anyone else present. *Yes/No* (skip next 2 question)
- How much time did you **spend at home by yourself yesterday?**
- How did you feel when you were **at home by yourself yesterday**? Rate each feeling on a scale from 0—did not experience at all—to 6—the feeling was extremely strong.
  - Matrix showing: *Happy/Interested/Frustrated/Sad*

#### Additional module

Overall, how did you feel **yesterday**? Rate each feeling on a scale from 0—did not experience at all—to 6—the feeling was extremely strong.

Matrix showing*: Happy/Interested/Frustrated/Sad/Enthusiastic/Content/Angry/Tired/Stressed/Lonely/Worried/Bored/Pain/Depressed/Joyful*

### Experienced Questions: Gallup Well-Being Index

- Did you experience **anger** during a lot of the day yesterday? *Yes/No*
- Did you experience **depression** during a lot of the day yesterday? *Yes/No*
- Did you experience **enjoyment** during a lot of the day yesterday? *Yes/No*
- Did you experience **happiness** during a lot of the day yesterday? *Yes/No*
- Did you experience **sadness** during a lot of the day yesterday? *Yes/No*
- Did you experience **stress** during a lot of the day yesterday? *Yes/No*
- Did you experience **worry** during a lot of the day yesterday? *Yes/No*

- Now, please think about yesterday, from the morning until the end of the day. Think about where you were, what you were doing, who you were with, and how you felt. **Did you learn or do something interesting yesterday**? *Yes/No*

- Now, please think about yesterday, from the morning until the end of the day. Think about where you were, what you were doing, who you were with, and how you felt. **Did you smile or laugh a lot yesterday**? *Yes/No*

- Now, please think about yesterday, from the morning until the end of the day. Think about where you were, what you were doing, who you were with, and how you felt. **Were you treated with respect all day yesterday**? *Yes/No*

- Now, please think about yesterday, from the morning until the end of the day. Think about where you were, what you were doing, who you were with, and how you felt. **Would you like to have more days just like yesterday**? *Yes/No*

#### Additional module

- Did you experience **enthusiasm** during a lot of the day yesterday? *Yes/No*
- Did you experience **contentment** during a lot of the day yesterday? *Yes/No*
- Did you experience **frustration** during a lot of the day yesterday? *Yes/No*
- Did you experience **fatigue** during a lot of the day yesterday? *Yes/No*
- Did you experience **loneliness** during a lot of the day yesterday? *Yes/No*
- Did you experience **boredom** during a lot of the day yesterday? *Yes/No*
- Did you experience **pain** during a lot of the day yesterday? *Yes/No*

What time did you wake up **yesterday**?: …..:……
What time did you go to bed **yesterday**?: …..:……

- Did you feel well-rested **yesterday morning** (that is, you slept well the night before)? *Yes/No*
- Was yesterday a normal day for you or did something unusual happen? *Yes, just a normal day/No, my day included unusual bad (stressful) things/No, my day included unusual good things*

Please think about the **things you did yesterday**. How did you spend your time and how did you feel?

- Yesterday, did you **watch TV**? *Yes/No* (skip next question)
- How much time did you spend **watching TV yesterday**?
- Yesterday, did you **work or volunteer**? *Yes/No* (skip next question)
- How much time did you spend **working or volunteering yesterday**?
- Yesterday, did you go for a **walk or exercise**? *Yes/No* (skip next question)
- How much time did you spend **walking or exercising yesterday**?
- Yesterday did you do any **health-related activities other than walking or exercise**? For example, visiting a doctor, taking medications or doing treatments. *Yes/No* (skip next question)
- How much time did you spend doing **health-related activities yesterday**?
- Yesterday did you **travel or commute**? E.g. by car, train, bus etc. *Yes/No* (skip next question)
- How much time did spend **travelling or commuting yesterday?**
- Yesterday did you **spend** **time with friends or family**? *Yes/No* (skip next question)
- How much time did you spend **with friends or family yesterday?**
- Yesterday did you **spend time at home by yourself**? Without a spouse, partner or anyone else present. *Yes/No* (skip next question)
- How much time did you **spend at home by yourself yesterday?**

- **[2 activities reported by the respondent were randomly selected for the ELSA experienced affect module. For example, if activity “walking or exercising was chosen”, the question was:]**

- How did you feel when you were **walking or exercising**? Rate each feeling on a scale from 0—did not experience at all—to 6—the feeling was extremely strong.

- Matrix showing: *Happy/Interested/Frustrated/Sad*

### Experienced Questionnaire: HWB-12

Now we would like you to think about yesterday. What did you do yesterday and how did you feel?

To begin, please tell me what time you woke up **yesterday**: …………
And what time did you go to sleep **yesterday**?: …………

Now please take a few quiet seconds to recall your activities and experiences *yesterday*

Good, now I have questions about your experiences **yesterday**. **[Randomized order of emotions]**

- Yesterday, did you feel **happy**? Would you say: *Not at all/A little/Somewhat/Quite a bit/Very*
- Yesterday, did you feel **enthusiastic**? Would you say: *Not at all/A little/Somewhat/Quite a bit/Very*
- Yesterday, did you feel **content**? Would you say: *Not at all/A little/Somewhat/Quite a bit/Very*
- Yesterday, did you feel **angry**? Would you say: *Not at all/A little/Somewhat/Quite a bit/Very*
- Yesterday, did you feel **frustrated**? Would you say: *Not at all/A little/Somewhat/Quite a bit/Very*
- Yesterday, did you feel **tired**? Would you say: *Not at all/A little/Somewhat/Quite a bit/Very*
- Yesterday, did you feel **sad**? Would you say: *Not at all/A little/Somewhat/Quite a bit/Very*
- Yesterday, did you feel **stressed**? Would you say: *Not at all/A little/Somewhat/Quite a bit/Very*
- Yesterday, did you feel **lonely**? Would you say: *Not at all/A little/Somewhat/Quite a bit/Very*
- Yesterday, did you feel **worried**? Would you say: *Not at all/A little/Somewhat/Quite a bit/Very*
- Yesterday, did you feel **bored**? Would you say: *Not at all/A little/Somewhat/Quite a bit/Very*
- Yesterday, did you feel **pain**? Would you say: *Not at all/A little/Somewhat/Quite a bit/Very*

#### Additional module

**[Randomized order of emotions]**

- Yesterday, did you feel **depressed**? Would you say: *Not at all/A little/Somewhat/Quite a bit/Very*
- Yesterday, did you feel **joyful**? Would you say: *Not at all/A little/Somewhat/Quite a bit/Very*
- Yesterday, did you **learn or do something interesting**? Would you say: *Not at all/A little/Somewhat/Quite a bit/Very*
- Did you feel well-rested **yesterday morning** (that is, you slept well the night before)? *Yes/No*
- Was **yesterday** a normal day for you or did something unusual happen? *Yes, just a normal day/No, my day included unusual bad (stressful) things/No, my day included unusual good things*

Please think about the **things you did yesterday**. How did you spend your time and how did you feel?

- Yesterday, did you **watch TV**? *Yes/No* (skip next question)
- How much time did you spend **watching TV yesterday**?
- Yesterday, did you **work or volunteer**? *Yes/No* (skip next question)
- How much time did you spend **working or volunteering yesterday**?
- Yesterday, did you go for a **walk or exercise**? *Yes/No* (skip next question)
- How much time did you spend **walking or exercising yesterday**?
- Yesterday did you do any **health-related activities other than walking or exercise**? For example, visiting a doctor, taking medications or doing treatments. *Yes/No* (skip next question)
- How much time did you spend doing **health-related activities yesterday**?
- Yesterday did you **travel or commute**? E.g. by car, train, bus etc. *Yes/No* (skip next question)
- How much time did spend **travelling or commuting yesterday?**
- Yesterday did you **spend** **time with friends or family**? *Yes/No* (skip next question)
- How much time did you spend **with friends or family yesterday?**
- Yesterday did you **spend time at home by yourself**? Without a spouse, partner or anyone else present. *Yes/No* (skip next question)
- How much time did you **spend at home by yourself yesterday?**

- **[2 activities reported by the respondent were randomly selected for the ELSA experienced affect module. For example, if activity “walking or exercising was chosen”, the question was:]**

- How did you feel when you were **walking or exercising**? Rate each feeling on a scale from 0—did not experience at all—to 6—the feeling was extremely strong.
  Matrix showing: *Happy/Interested/Frustrated/Sad*

## Footnotes

1 The Positive and Negative Affect Schedule (PANAS) is another example of a scale that elicits experienced well-being aggregated over a number of episodes, e.g. past few days, past few weeks, past month, etc. See for instance, Watson et al. (1988), or Watson and Clark (1994). We restrict our discussion to the measures that are closely related to the measures included in HRS, SHARE, and ELSA. These all only elicit experienced well-being over the past day.

2 For instance, Watson and Clark (1994) show that self-ratings on their affect scales correlate with peer ratings; Evaluative well-being measures have been shown to correlate with income, health, social contacts, and job satisfaction (e.g. Kapteyn et al. 2010; Van Praag et al. 2003; Layard 2005).

3 See “Appendix” for detailed questionnaires.

4 Including the two experienced and one eudemonic well-being questions taken from ONS.

5 Factor analysis aims at explaining covariances and correlations between many variables in terms of linear functions of a few underlying latent variables called factors (Bollen 1989; Morrison 1990).

6 Typically, the number of factors is limited by selecting a cutoff point for eigenvalues (typically, one), as their magnitude is associated with their capability to “account for” the correlations in the observed variables. A sharp drop in the size of eigenvalues can also be used to inform the choice in the number of factors (Bollen 1989). We make use of both techniques here.

7 When including those variables in our regressions, we do find some effect of major life events on evaluative subjective well-being measures (and hardly on experienced well-being), but only minor changes in the coefficients for current socio-economic and demographic variables (in particular, unemployment). So from the viewpoint of affecting the current findings regarding the effects of socio-economic and demographic variables, omitting major life events does not seem to matter very much. We intend to conduct a thorough analysis of the effect of life events in a separate paper.

8 Alternatively, we could have estimated a Random Effects model; the results of that specification are virtually indistinguishable from the results we obtain with the current specification. Since the dependent variables are not always continuous, we could also have used models for limited dependent variables, such as Ordered Logit or Ordered Probit. The advantage of using OLS throughout lies in the ease of interpretation, while it also facilitates comparison across the different scales. In practice results obtained by OLS often are very similar to those obtained by more complex methods.

9 To give an example: if we double the scale of a dependent variable, then all coefficients would double as well, but their ratios would of course stay the same.

10 Factor analysis of the Diener items yields one factor with eigenvalue >1 (the eigenvalue equals 3.69).
